# Correlation between biochemical, ultrasonographic and demographic parameters with ovarian response to IVF/ICSI treatments in Mexican women

**DOI:** 10.5935/1518-0557.20200040

**Published:** 2021

**Authors:** Alfredo Cortés Vázquez, José Modesto Alfredo Góngora Rodríguez, Alfredo Leonardo Cortés Algara, Jesús Daniel Moreno García

**Affiliations:** 1 Centro Médico Nacional 20 de Noviembre, Mexico City, Mexico

**Keywords:** IVF, ovarian, response, parameters, mexican, prediction

## Abstract

**Objective:**

Ovarian response from a conventional ovarian stimulation protocol is a crucial step in IVF/ICSI treatments. This ovarian response encompasses a wide range of outcomes at the extremes, leading to either excessive responses with the risk of life-threatening conditions like ovarian hyperstimulation syndrome (OHSS), or poor ovarian response (POR) with poor outcomes. This study aims to integrate biochemical, ultrasonographic and demographic parameters into a mathematical formula able to predict ovarian response to stimulation in IVF/ICSI in gonadotropin-releasing hormone (GnRH) antagonist protocols.

**Methods:**

This retrospective analysis included 147 patients submitted to an ovarian stimulation protocol combining recombinant FSH and gonadotropin-releasing hormone antagonist. All the parameters were correlated with the Spearman Rho and Pearson´s correlation coefficient. Once the data was normalized, we used the multiple linear regression models, checking the results with the progressive discriminating analysis.

**Results:**

We classified the database according to the correlation with the number of oocytes retrieved; the progressive discriminating analysis resulted in the following equation: oocytes retrieved = 2.312-0.130 (FSH) + 0.562 (AFC).

**Conclusions:**

The incorporation of 2 ovarian reserve parameters into a regression equation enables knowing the number of retrieved oocytes in each patient with 80.5% sensitivity and 55.4% specificity.

## INTRODUCTION

Today, it is undeniable that *in vitro fertilization* (IVF) offers the highest per-treatment success rate for infertile patients. Even though an estimated 7 million couples suffer from infertility, only 3% of these patients have access to an IVF treatment ^(Choi et al., 2013)^. There are several reasons for this underutilization, such as high cost, limited insurance reimbursement and success with other treatments. Since the early 1900s, several studies characterized the pituitary regulation of gonadal function, which is the basis for developing gonadotropin preparations for ovarian stimulation ^(Alper & Fauser, 2017)^.

Ovarian stimulation with gonadotropins is a crucial step in intracytoplasmatic sperm injection (ICSI) treatments. Ovarian stimulation aims at recruiting multiple follicles to have many oocytes and increase the chances of pregnancy in IVF ^(Sighinolfi et al., 2018)^. This ovarian response encompasses a wide range of outcomes, at the extremes it may lead to either excessive responses with the risk of life-threatening conditions, such as ovarian hyperstimulation syndrome (OHSS), or poor ovarian response (POR) with low results ^(Chalumeau et al., 2018)^. Therefore, we need fertility treatment customization to avoid IVF/ICSI cycle cancellation due to inadequate responses to gonadotropins. An individualized dosing regimen may decrease the risk of moderate or even severe cases of OHSS, as well as the incidence of preventive interventions ^(Fernández-Sánchez et al., 2019)^.

Ovarian response to stimulation with gonadotropins is linked to ovarian reserve (OR), which is defined as a woman´s reproductive potential, this potential is determined by the quantity and quality of the oocytes ^(Podfigurna et al., 2018)^. The ovarian reserve is a complex phenomenon that is affected by age, genetics and various environmental interactions ^(Tal & Seifer, 2017)^. This assessment is the key to establishing prognosis and to choose the most adequate ovarian stimulation protocol to apply. In clinical practice, physicians often rely on their clinical experience and judgment when selecting an appropriate starting dose of follicle-stimulating hormone (FSH) ^(van Tilborg et al., 2012)^. Accurate knowledge of OR can help physicians use more patient-friendly ovarian stimulation protocols in older patients, since no difference was seen in terms of cumulative pregnancy rates between conventional and mild stimulation protocols ^(Alper & Fauser, 2017)^. Since hormonal stimulation is the most expensive part of IVF and ICSI treatments ^(van Tilborg *et al*., 2012)^, accurate predictive models can provide more cost-effective strategies. Cost-effective treatments in women who are eligible for IVF or ICSI treatment are important in modern society since many women delay childbearing.

Ovarian reserve can be appraised mainly by 2 direct parameters, which are: Antral Follicle Count (AFC) and Anti-müllerian hormone (AMH). These parameters have been reported to have the highest predictive value concerning the ovarian response ^(Chalumeau et al., 2018)^. There are indirect parameters, like age and FSH levels, that influence the ovarian response to gonadotropin stimulation protocols. On the other hand, it is also well established that body mass index (BMI) can have some influence on the ovarian response to the IVF/ICSI treatments. It is essential to know that there is a strong positive age-independent relationship between AMH and the ratio of euploid blastocysts ^(La Marca *et al*., 2017)^.

Therefore, it is vital to have in mind that ovarian response seems to be multifactorial, and hence, it is necessary to consider all the parameters, so as to make the right initial dose decision. This study is aimed to integrate biochemical, ultrasonographic and demographic parameters in a mathematical formula, able to predict the ovarian response to stimulation from IVF/ICSI in gonadotropin-releasing hormone (GnRH) antagonist protocols.

## MATERIAL AND METHODS

### Patients

We performed a retrospective study, where we analyzed 147 patients, who met all the inclusion criteria and underwent their first ovarian stimulation protocol, combining recombinant FSH and gonadotropin-releasing hormone antagonist in 2018, at the Reproductive Endocrinology Department at Centro Medico Nacional 20 de Noviembre.

The patients’ characteristics are summarized in Table 1. Patients were included in the study if the delay between the OR evaluation (AFC, FSH, estradiol) and IVF/ICSI was less than a year. Attempts in which the follicle puncture appeared difficult were excluded from the study.

**Table 1:** Demographic data

Demographic data	
N	147
Age	35.6±3.6
FSH* (IU/l)	6.7±3.9
Estradiol (pg/ml)	47.7±26.9
BMI^†^(kg/m^2^)	25.8±3.4
AFC^‡^	9.7±4.0
Total number of injected rFSH units	2383± 614.5
Stimulation length	9.6±1.3
Number of collected oocytes	6.9±4.7

FSH*=Follicle Stimulating Hormone.BMI^†^=Body Mass Index,AFC^‡^=Antral Follicle Count., Values expressed as mean±SD unless otherwise indicated.

OR was evaluated by AFC (2-9mm using a 2D 7.5 MHz vaginal probe), all hormone measurements (FSH, estradiol) were conducted in the same laboratory (Reproductive Endocrinology Department at Centro Médico Nacional 20 de Noviembre), using the same methods, between cycles on days 2 and 3.

We collected the data from the Reproductive Endocrinology Department database. The Centro Medico Nacional 20 de Noviembre Ethics Committee approved this study.

### Ovarian stimulation

All the patients were stimulated with Gonal F (150 to 450 UI follitropin alfa, Merck Serono, Switzerland) subcutaneously. The physicians subjectively chose the daily rFSH starting dose according to age, BMI and OR. On stimulation day 6, we adapted the doses according to the results of ovulation monitoring (ultrasonographic evaluation), and started Cetrotide (cetrorelix, 0.250mg, Merck Serono, France). Ovulation was triggered when at least 3 or more follicles reached an 18-20 mm diameter, with Ovidrel (Corio gonadotropin 250 micrograms, Merck Serono, Italy) subcutaneously. Oocytes retrieved from the follicles >12 mm in diameter were transvaginally retrieved under ultrasound guidance 36 hours later. We assessed ovarian response through the number of recovered oocytes.

### Statistical Analysis

All the parameters were correlated with the Spearman Rho and Pearson´s correlation coefficient. After normalizing the data, we used the multiple linear regression model, checking the results with the progressive discriminating analysis. In every contrast, we chose a *p*-value < 0.05.

## RESULTS

147 patients underwent an IVF/ICSI treatment; we summarized the patients’ characteristics in Table 1. From the total parameter measurements, only weight, size and BMI had a normal distribution. The distributions of the other parameters (including age) were not normal. Considering the distribution of these parameters, they were correlated with the Spearman Rho correlation coefficient, and there were significant negative correlations between the number of oocytes retrieved and FSH levels (rho= -0.236, *p*=0.004). This evidence shows that higher levels of basal FSH were associated with a lower number of retrieved oocytes (Graphic 1). Meanwhile, the number of retrieved oocytes had a stronger and positive correlation with the AFC values (rho=0.541, *p*=0.0001). In this case, higher AFC values were associated with a higher number of retrieved oocytes (Graphic 2).

**Graphic 1:**
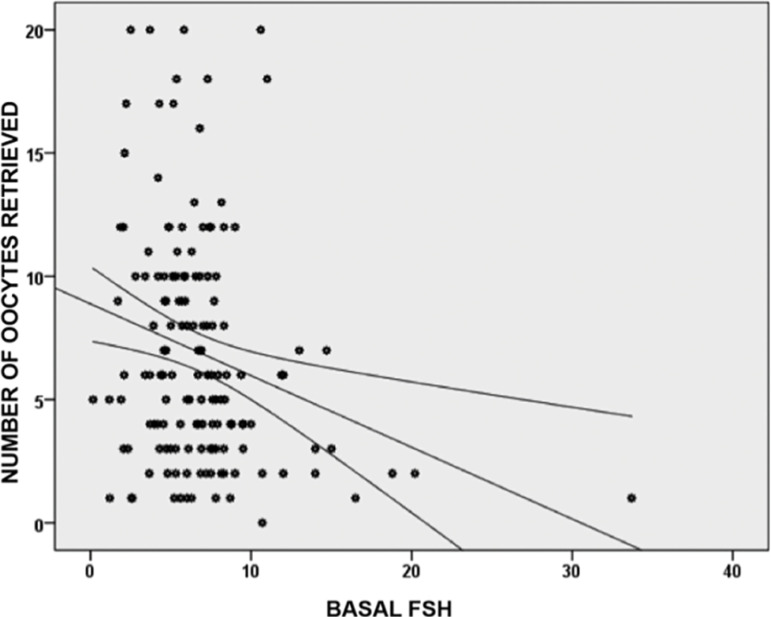
Correlation between Basal FSH levels and the number of oocytes retrieved for 147 patients. Higher Basal FSH values were negatively correlated with the number of oocytes retrieved, with a Spearman´s Rho negative coefficient of -0.236

**Graphic 2:**
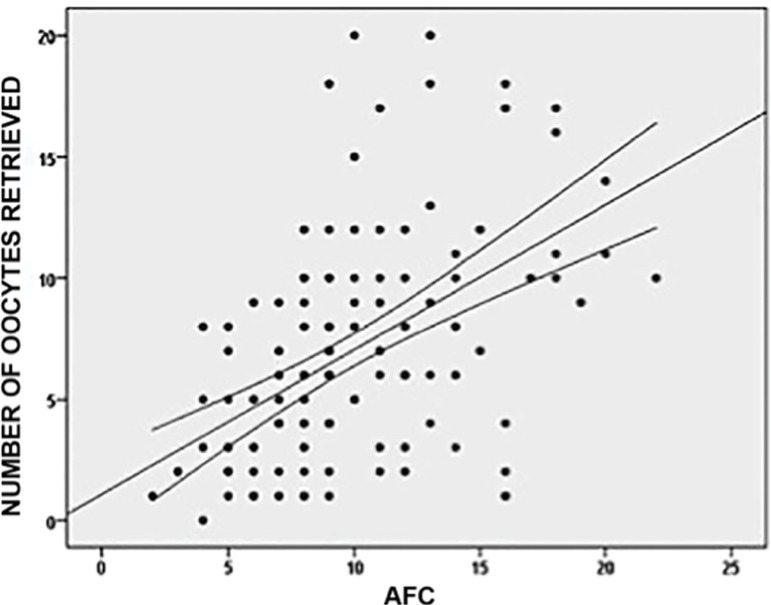
Correlation between Antral Follicle Count (AFC) and the number of oocytes retrieved. Higher AFC values were positively correlated with a higher number of oocytes retrieved. This parameter had a positive Spearman’s Rho correlation of 0.541

As shown in Table 2, the parameters showing the lowest correlation with the ovarian response are:

**Table 2:** Correlation between ovarian response (number of collected oocytes) and the different demographic, ultrasonographic and biochemical parameters

Parameters	Number of collected oocytes
	Spearman Rho´s Coefficient
Age	-0.122
Weight	0.050
Height	0.003
BMI*	0.043
Basal estradiol	0.109
AFC^†^	0.541
Basal FSH^‡^	-0.236
Stimulation length	0.049
Total gonadotropin dosage	-0.098

BMI*=Body Mass Index, ^†^AFC=Antral Follicle Count, ^‡^FSH=Follicle Stimulating Hormone.

1. Age
2. Weight
3. Height
4. BMI
5. Basal estradiol
6. Total stimulation days
7. Total gonadotropin dosage

Furthermore, basal FSH and AFC were negatively correlated with a rho coefficient of -0.294 (*p*=0.0001). Therefore higher basal FSH values are correlated with lower AFC values (Graphic 3).

**Graphic 3:**
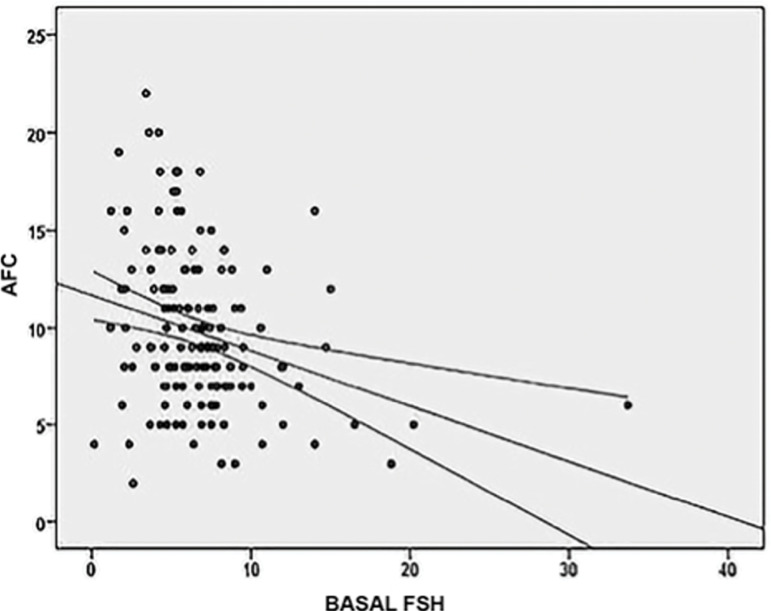
When basal FSH levels and AFC values are correlated, there was a negative interrelation among them. Higher FSH levels were correspondent with lower AFC values, with a statistically significant Spearman’s rho coefficient of -0.294 (*p*=0.0001)

Additionally, basal FSH values were positively correlated with the patient`s weight, with a positive rho of 0.234 (*p*=0.004), meaning that the higher the patient´s weight, the higher the basal FSH values they had (Graphic 4).

**Graphic 4:**
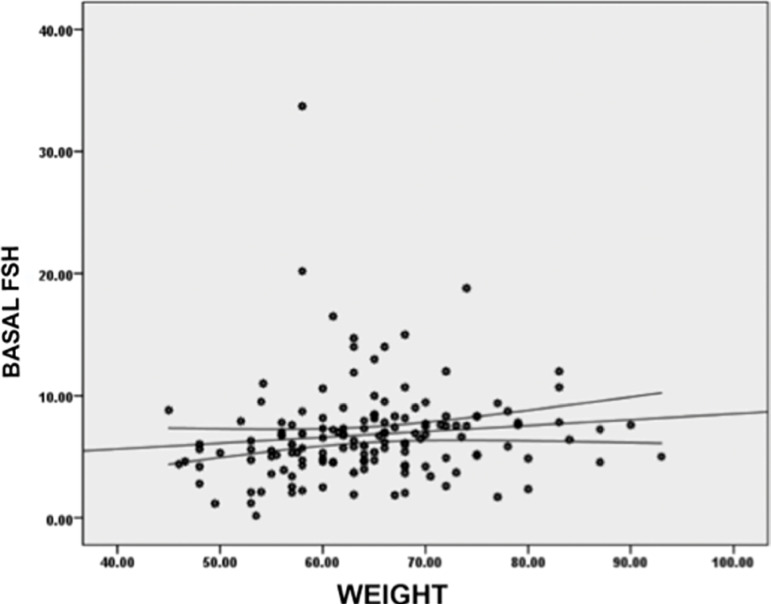
Correlation between patient’s weight and basal FSH levels. Higher FSH values had a positive correlation with a higher patient’s weight. This correlation had a statistically significant Spearman’s Rho coefficient of 0.234 (*p*=0.004)

Moreover, there was colinearity between basal FSH values and AFC values; therefore, the multiple linear regression considered only the AFC values as significant, even when they were transformed with the natural logarithm to normalize them. The multiple correlation coefficient was 0.516 (*p*=0.0001), and the equation for the prediction would be the one presented in Table 3.

**Table 3:** Coefficients*

Model	Non standardized coefficients		Categorized Coefficients	T	Sig.
	B	Error tip.	Beta		
1 (Constant) BASAL FSH^†^ AFC^‡^	2.312	1.231		1.878	.062
	-.130	.090	-.108	-1.447	.150
	.562	.088	.475	6.391	.000

* Dependent variable: OOCYTES RETRIEVED,

† FSH=Follicle Stimulating Hormone,

‡ AFC=Antral Follicle Count

### Number of oocytes retrieved= 2.312- 0.130 (basal FSH) + 0.562 (AFC)

Now, when we stratified our population into 3 groups, according to their weight (maximal weight - minimal weight/3) for a 16 kg amplitude, we found very significant differences in the AFC, FSH, total gonadotropin dosage and median values on stimulation days.

It is essential to notice that the AFC median values decrease as weight increases (Table 3). Meanwhile, FSH total stimulation days and total gonadotropin dosage increase as weight increased. It should be pointed out that there was no difference in the number of oocytes retrieved among the different weight groups.

We adjusted the equation to get a better ovarian response prediction according to weight. In Table 4, we compared median values for each parameter according to weight stratum.

**Table 4:** Median comparison according to weight stratification

Weight stratum (number of cases)	AFC*	Basal FSH^†^	Total gonadotropin dosage	Stimulation length
45-60 (n=53)	10.6±4.1	6.1±4.9	2331.3±600.4	9.6± .6
61-76 (n=78)	9.2±3.9	7.2±3.2	2350.3±583.2	9.5±1.2
77-93 (n=16)	8.8±3.6	6.8±2.7	2714.0±740.0	10.3±1.0
*p* Anova	0.09	0.07	0.02	0.10
*p* H Kruskal-Wallis	0.10	0.08	0.01	0.05

* AFC=Antral Follicle Count,

† FSH=Follicle Stimulating Hormone

After we compared the median values for the different parameters, we made the correlations for each weight class, and the multiple linear regression model coefficient, and each predictive equation was adjusted for each weight class. Table 5 summarizes the weight-stratified predictive equations.

**Table 5:** Number of retrieved oocytes predictive equations according to weight class

Weight stratum	Predictive equation
45-60 (n=53)	1.435 - 0.040 (Basal FSH*) + 0.560 (AFC^†^)
61-76 (n=78)	3.562 - 0.293 (Basal FSH) + 0.566 (AFC)
77-93 (n=16)	1.808 - 0.053 (Basal FSH) + 0.631 (AFC)

* FSH=Follicle Stimulating Hormone.

† AFC=Antral Follicle Count

Also, we calculated the sensitivity and specificity of our model, resulting in 80.5% and 55.4% specificities, respectively.

## DISCUSSION

Every ovarian stimulation protocol aims at producing a high-quality oocyte cohort, avoiding an excessive number of follicles ^(La Marca et al., 2017)^. This is imperative, because the number of retrieved oocytes is a critical prognostic factor in assisted reproductive techniques due to increasing medical literature stating that there is an optimal number of retrieved oocytes, instead of maximal oocytes number as a result of controlled ovarian stimulation when one desires a fresh embryo transfer ^(Papaleo et al., 2016)^.

The live birth rates continuously increase when we retrieve between 8 to 14 oocytes. It is due to the number of embryo/blastocysts available for transfer, considering the gonadotropin dosage, kind of stimulation (minimal vs. conventional) or each patient's profile ^(Arce et al., 2014)^.

With the goal of having an optimal number of oocytes available, the selection of a proper initial gonadotropin dosage for each patient is, therefore, the most crucial clinical decision. However, the initial dosing is based mainly on the physician´s experience, and many other parameters like previous ovarian stimulations, age, and other ovarian reserve markers ^(Papaleo et al., 2016)^. The new challenge for physicians is to identify each patient´s phenotype, to choose a better and more individualized IVF treatment from the first cycle ^(Nardo et al., 2011)^.

There are a limited number of models proposed to establish the initial gonadotropin dosing in controlled ovarian stimulation. One of the first was proposed by Popovic-Todorovic, which includes 5 parameters like age, tobacco use, testosterone concentrations, Doppler score and AFC ^(Popovic-Todorovic *et al*., 2003a^; ^2003b^; ^Olivennes, 2008)^. Afterwards, other models appeared, such as CONSORT, PIVET and a nomogram developed by ^La Marca & Sunkara (2014)^, which have the advantage to being readily available.

A recently published prospective study found that individualization of the gonadotropin dosage allowed a 50% reduction in the incidence of OHSS in the study group ^(Fernández-Sánchez et al., 2019)^ with a statistically significant difference. They also found a reduction in the need to implement preventive measures, compared to a conventional stimulation regimen ^(Fernández-Sánchez *et al*., 2019)^. Some authors found that a nomogram could provide a better calculation of the initial gonadotropin dosage, enabling the use of a minimally effective dosage for every patient and donor ^(Papaleo et al., 2016)^. This approach could significantly decrease the rate of suboptimal patients ^(Allegra et al., 2017)^.

However, it is vital to take into consideration that some of the exclusion criteria used in some of the studies, like the CONSORT, limit their implementation and generalized use ^(Olivennes et al., 2015)^. Some of the exclusion criteria used are poor ovarian response in 2 previous IVF cycles, age less than 35 years, history of OHS, PCOS, among others, and also the use of an agonist ovarian stimulation protocol, which has fallen in disuse.

We found the same trend (like other authors) concerning AFC behavior and its association with the number of oocytes retrieved, in addition to the negative correlation with FSH ^(Allegra et al., 2017)^. However, as opposed to ^Baker et al. (2018)^ we did not find any influence of age in the number of oocytes retrieved after a controlled ovarian stimulation.

Concerning the prediction of POR, we found that AMH has a higher sensitivity and specificity than FSH and AFC together ^(Baker et al., 2018)^, although other authors reported that AFC and AMH have a similar correlation for the prediction of POR ^(Alanazi et al., 2018)^, as well as higher FSH levels, which have a correlation with low AMH concentrations with POR.

As for the gonadotropin dosage used and stimulation duration, in our study we did not find any influence in the oocyte`s retrieval rate, unlike authors like ^Arce et al. (2014)^, who found that there is a direct relationship between gonadotropin dosage and the number of oocytes retrieved. In the same way, the increase in gonadotropin dosage is associated with fewer stimulation days ^(Arce *et al*., 2014)^. It is essential to mention that in the same study, there was a lower fertilization rate and a lower blastocyst rate, in an inverse relationship with gonadotropin dosage ^(Arce *et al*., 2014)^. This data is consistent with the findings from ^Labarta et al. (2018)^, who found that patients with POR who underwent a mild ovarian stimulation protocol had higher fertilization rates, mature oocytes and higher-good quality embryos, compared to conventional stimulation protocols. This finding could explain in part why the cumulative pregnancy rate has shown to be comparable between conventional and mild stimulation protocols in older patients ^(Alper & Fauser, 2017)^.

## CONCLUSIONS

The integration of 2 ovarian reserve markers in a formula, allows knowing the number of retrieved oocytes for each patient. It is essential to point out the lack of more prospective studies to validate the formula so it could be applied systematically.
